# When immune landscapes diverge: How immune cell heterogeneity shapes cancer immunotherapy response

**DOI:** 10.47248/chp2603030009

**Published:** 2026-07-10

**Authors:** Jessie L. Chiello, Nijamuddin Shaikh, AJ Robert McGray

**Affiliations:** 1 Department of Immunology, Roswell Park Comprehensive Cancer Center, Buffalo, NY 14263, USA; 2 Department of Gynecologic Oncology, Roswell Park Comprehensive Cancer Center, Buffalo, NY 14263, USA

**Keywords:** Tumor immune heterogeneity, Bispecific T cell engager, Adoptive T cell therapy, Multi-omics, Immune Checkpoint Blockade, Immunotherapy Response

## Abstract

Amplifying the antitumor immune response using immunotherapy has significantly improved patient survival and transformed cancer care. Findings from preclinical studies as well as clinical observations have firmly established that the intratumoral accumulation of T cells often serves as a positive predictor of immunotherapy response. While this observation has led to the commonly used “cold or hot” classifiers to describe the T cell landscape within a tumor, this is increasingly being recognized as an oversimplification. Instead, there is a growing appreciation for the broader composition, spatial localization, and dynamic nature of the tumor immune landscape across cancer types. In this perspective, we describe the diverse and unique immune correlates that have been associated with response (or lack thereof) following immunotherapy. Beginning with our own published findings related to the heterogeneous immune landscapes associated with divergent responses to adoptive T cell therapy combined with immune checkpoint inhibition, we then overlay key observations from other recently published studies to highlight the impact of the broader immune landscape in determining treatment outcome. Finally, we recommend leveraging modern multi-omic analysis tools prior to and during immunotherapy treatment to define immune signatures associated with favorable response, those that can predict treatment outcome, or which can be leveraged to identify complementary therapeutic approaches.

## 1. The Immune Landscape of Cancer: Moving Beyond ‘Cold’ *Versus* ‘Hot’ Classifiers

Immunotherapies have revolutionized cancer treatment, leading to remarkable response durability and improved survival for many cancer patients. Despite this paradigm shift in cancer care, the benefit of immunotherapy is observed in only a subset of patients while others fail to respond [1,2]. Although various factors including age, cancer type and disease burden, number of metastatic sites, molecular alterations, and prior lines of therapy have been identified as potential determinants of immunotherapy response, the immune cell composition of the tumor microenvironment (TME) dramatically impacts therapeutic outcome. While earlier work focused on categorizing tumors into ‘cold’ or ‘hot’ phenotypes based on tumor infiltrating lymphocyte (TIL) abundance (Figure 1A), this is now considered to be an oversimplification of the nuanced immune niches and diverse cell subsets found in the broader tumor immune landscape [3]. As such, an improved understanding of the complex immune composition of the TME, how the immune landscape of cancer shifts over time or in response to therapy, as well as the spatial organization of intratumoral immune cells is needed to enhance our capacity to rationally select immunotherapies for cancer patients. In this perspective, we aim to explore how immune profiling of the TME at baseline and on treatment can provide crucial insights related to mechanisms of treatment response or failure, as well as how studying immune cell heterogeneity within and across cancers can be therapeutically leveraged. Focusing on recently published work from our group and others, we explore phenotypic, functional, and spatial determinants associated with immunotherapy response, which have the potential to identify improved predictive biomarkers and to better select tailored therapeutic options. Collectively, our goal is to provide an actionable framework for integrating baseline and on-treatment data that effectively captures both immune cell complexity and dynamic changes in the TME. Potential limitations and challenges in implementing these approaches, opportunities to use new knowledge to inform mechanistic hypotheses or novel therapeutic strategies, as well as how this information can improve patient outcomes will also be discussed.

## 2. Shifts in the Tumor Immune Landscape Arise During Divergent Therapeutic Responses

In our recent publication [4], we evaluated if adoptive transfer of T cells engineered to secrete folate receptor alpha (FRα)-targeted bispecific T cell engagers (FR-B T cells) could rationally combine with immune checkpoint blockade (anti-PD-1) to treat ovarian cancer (OC). We first tested FR-B T cells ± anti-PD-1 against primary OC patient specimens using an *in vitro* co-culture system. Here, 17/20 tested OC samples responded to FR-B T cell therapy (based on IFN-γ production), while only 8 of these 17 samples demonstrated an enhanced response upon anti-PD-1 addition. Categorizing these as either responders (R) or enhanced responders (R+), we interrogated potential phenotypic drivers of R versus R+. While no difference in OC FRα positivity, tumor/immune cell PD-L1 levels, or differences in the overall CD45+ immune cell abundance in the TME were observed between R and R+ specimens, R specimens were found to have a >2.5-fold increase in CD206+ CD14+ myeloid cells (consistent with an immunosuppressive phenotype) compared to R+ specimens. As this finding was consistent with a role for the broader OC immune landscape in impacting the FR-B T cell + anti-PD-1 response, we next utilized an immunocompetent OC model to globally assess the cellular changes in the OC TME during FR-B T cells ± anti-PD-1 treatment. Similar to our observations in clinical OC samples, FR-B T cells + anti-PD-1 produced divergent treatment responses. Specifically, a subset of animals receiving combination therapy developed early progressive disease (PD), whereas approximately 50% of mice treated with FR-B T cells + anti-PD-1 achieved durable response (DR). Using single cell transcriptomics (scRNA-seq) during the acute phase of treatment response and again once PD and DR phenotypes had emerged, we observed that while FR-B T cells + anti-PD-1 led to rapid (and nearly complete) clearance of FRα+ OC cells, divergent immune landscapes developed between PD and DR. While PD mice showed limited accumulation of predominantly naïve T and B cells and had high frequencies of Arg1+ macrophages and increased neutrophils, DR mice were characterized by accumulation of activated B cells, increased effector memory CD4+ T cells, and Cxcl13-expressing macrophages. Of note, FR-B T cells were no longer readily detected in the OC TME at the time of PD/DR divergence, suggesting not only a shift towards endogenous antitumor responses over time, but also distinct immune trajectories impacting response durability.

## 3. Heterogeneous Immune Landscapes Impact Treatment Outcome Across Tumor Indications

Our observations in OC related to dynamic changes in the tumor immune composition following immunotherapy are not unique and multiple published studies have now demonstrated that the accumulation of distinct pro-inflammatory or immunosuppressive cell types correlates with treatment success or failure [5–7]. In order to contextualize these observations and begin to define the generalizable immune features or principles associated with improved therapeutic outcome across cancers, this section highlights other recently published studies identifying distinct immune features associated with immunotherapy response. Of note, recent advances in experimental models, exciting new developments in flow cytometry, multiplex imaging, and multi-omic analysis, and the emergence of next generation computational tools to integrate complex datasets have led to a deeper understanding of the cellular milieu underlying immunotherapy responses (Figure 1B). For example, Rosario & Long *et al*. [8] identified increased T cell and B cell infiltrates along with elevated levels of T and B cell activation, differentiation, and proliferation in OC patients that achieved durable clinical benefit (DCB) following combination immunotherapy. Additionally, an increased tertiary lymphoid structure (TLS) gene signature (Ccl19, Cxcl13, Ccl21) and enrichment for lactic acid-producing gut bacteria were also associated with improved therapeutic outcome. Indeed, mature TLS (mTLS) are associated with improved survival in many tumor types, including OC and non-small cell lung cancer (NSCLC), and are predictive of therapeutic response [9,10]. Regarding TLS, MacFawn *et al*. [11] have demonstrated that the immune cell activity in the case of OC is highly dependent on the level of TLS maturity, wherein less mature TLS niches have low immune cell activity, contrasting with increased immune cell function within more-developed TLS. From a mechanistic standpoint, the presence of these more developed or mTLS provides a pro-inflammatory environment to support direct interaction between T cells, B cells, and antigen presenting cells (such as dendritic cells; DCs) to enhance antitumor responses, with the presence of mTLS associated with higher sensitivity to immunotherapies [12]. Importantly, these findings provide a potential framework to stratify patients for immunotherapy trials based on the presence of TLS.

Beyond TLS formation and downstream functionality, multiple reports have identified unique immune cell subsets and/or spatially-defined characteristics that are associated with patient outcome and/or treatment response. Hammerl *et al*. [13] identified three discrete spatial immunophenotypes (inflamed, excluded, and ignored) in breast cancer, with the presence of specific immune cell subsets effectively predicting divergent anti-PD-1 responses. Responders exhibited enrichment of CLEC9A+ DCs, high T cell receptor (TCR) clonality, elevated expression of T cell co-inhibitory receptors, and necrosis markers, while non-responders demonstrated high CD163+ myeloid cell density, collagen-10 deposition, enhanced glycolysis, and activation of TGF-β/VEGF pathways. Another breast cancer study carried out longitudinal tumor sampling during the course of immunotherapy, revealing the response to a PD-L1/CTLA-4 bispecific antibody in combination with a dual epitope blocking anti-HER2 bispecific antibody was associated with increased levels of CD8+ T cells, activated conventional type 1 and type 2 DCs, and reprogramming of macrophages towards an inflammatory state [14]. Looking more broadly at the impact of the tumor spatial microenvironment (TSME) across 12 different cancer types, Li *et al.* [15] identified multiple local cellular programs (LCP) and 13 recurrent cell-cell interacting niches, with each niche differentially impacting OS in patients. Stratifying patients across individual niches based on OS, the investigators found that Niche_4-like patients had the worst OS, whereas Niche_11-like patients had the longest OS, despite both niches being characterized by macrophage-related LCPs. Of note, Niche_4-associated macrophages were found in close proximity to tumor cells (M02 macrophages), whereas Niche_11 macrophages (M01 macrophages) were proximal to T cells. A secondary analysis compared proportions of M01 and M02, grouping macrophage states as G1 (M01^hi^M02^lo^), G2 (M01^hi^M02^hi^), G3 (M01^lo^M02^lo^), or G4 (M01^lo^M02^hi^) and overlaying these classifiers with survival outcomes. Across multiple indications (NSCLC, hepatocellular carcinoma, breast cancer), patients in G1 had better OS, whereas those categorized in G4 had reduced OS. Furthermore, analysis of pre-treatment samples from a cohort of melanoma patients treated with anti-PD-1 revealed that responders were enriched for M01 states (G1 and G2), whereas G4 trended towards increased abundance in non-responders, highlighting the distinct role of macrophages and their spatial distribution on prognosis and treatment outcomes.

Understanding cellular localization and tumor-host interactions can reveal key features related to the evolution of tumor metastasis, as well as mechanistic failure points of antitumor immune responses. Feng *et al.* [16] leveraged a multi-omic pipeline by combining paired whole genome and RNA sequencing samples, scRNA-seq, and high-resolution spatial transcriptomic data to interrogate tumor-host co-localization patterns in OC. Using the STARLETS framework (Spatiotemporal Tumor Clone Evolution Tracking System), they reported on bidirectional tumor-host interactions that can select for immune-evasive tumors, as well as the formation of tripartite structures containing SPP1+ macrophages, MMP11+ myofibroblastic cancer-associated fibroblasts (myCAFs), and epithelial cells, which formed at metastatic sites and in ascites. Targeting the SPP1-CD44 axis using a CD44 antagonist or a SPP1-neutralizing antibody reduced OC progression in preclinical studies and retrospective analysis of data from previously conducted clinical trials revealed that a higher abundance of SPP1+ macrophages was associated with responses to both oncolytic virotherapy and neoadjuvant poly(ADP-ribose) polymerase (PARP) inhibition, underscoring a potential for immune contexture to augment tumor sensitivity to treatment. In this regard, a recently published large-scale analysis of features associated with >10-year survival in OC patients highlighted that co-infiltration by diverse immune cell subsets including T cells, B cells, plasma cells, as well as CD68+PD-L1+ tumor-associated macrophages (TAMs) was increased in long-term survivors (LTS) when compared to short-term survivors (STS) [17]. Further, while immune infiltration was generally higher in epithelium-low compared to epithelium-high tumors, multiple immune cell subsets (including T cells, B cells, and plasma cells) were prognostically favorable in epithelium-high tumors and/or those with a C4/Differentiated molecular subtype, suggesting that the epithelial content of tumors and not simply the overall immune cell abundance represents a key determinant of outcome.

Genetic alterations including tumor mutation burden (TMB) and mismatch repair (MMR) status significantly impact immune checkpoint inhibitor (ICI) responses, however, a study in colorectal cancer (CRC) patients suggests that the tumor immune composition is also a key hallmark of ICI response in CRC. Immune correlates associated with robust ICI response were cytotoxic T and NK cells, and antigen presenting CXCL10+ TAMs, contributing to induction of a local interferon-high immunophenotype [18]. The proximity to IFN-producing cells in turn stimulates expression of CD74 on antigen presenting cells, suggesting the presence of CD74 can potentially be used as a predictor of an ICI-responsive immune environment. In NSCLC, expression of the glucose transporter SLC2A1 on TAMs hindered CD8+ T cell infiltration and consequently conferred resistance towards anti-PD-L1 therapy [19], further highlighting the complexity of TAM functionality in the TME.

Collectively, emerging evidence from multiple studies highlights how distinct immune compositions (either present at baseline or resulting from treatment) can dramatically influence immunotherapy responses. To this point, Ma *et al*. [20] argue in their recent perspective article that understanding the global TME, described as cellular neighborhoods, not only can provide a more detailed and standardized approach for characterizing the TME, but also allows for development of actionable and spatially-targeted treatment approaches, based on spatial location, composition, and functional state of the cellular infiltrate, which aligns with our own views. While many of the mechanisms that differentiate responders from non-responders following immunotherapy may be (i) cancer-type specific, (ii) include multifaceted and complex phenotypic/functional cell types, (iii) differ in the availability of key cytokines and chemokines, or (iv) diverge in either the composition of immune niches or cellular interactions (all of which may change dynamically over the course of therapy; Figure 1C), an emerging consensus suggests a predominance of antitumor T cells, proinflammatory myeloid cells, localized structures harboring mature T and B cells (such as TLS), and a tumor-restraining chemokine and cytokine profile are associated with immunotherapy responsiveness. Importantly, while these immune features have been associated with improved outcomes and/or enhanced immunotherapy response, further validation across large patient cohorts will be required to distill these findings into robust and actionable biomarkers, particularly if they are to be implemented in real time to support clinical decision making. Furthermore, while beyond the scope of this immune landscape-focused perspective, non-immune components of the TME can also impact immunotherapy response. For example, two distinct subsets of cancer associated fibroblasts (CAFs), one found in early-stage (MYH11+ αSMA+ CAFs) and one in late-stage (FAP+ αSMA+ CAFs) tumors, were associated with ICI resistance in a significant subset of mTLS-positive NSCLC patients [21]. These CAF populations can restrict T cell extravasation, mediate immune exclusion, elicit T cell exhaustion, and increase the abundance of CD4+ T regulatory cells in the TME. As such, detailed analysis of the entire cellular landscape of tumors is required to uncover mechanisms of therapeutic success or failure.

For patients who fail to respond to current immunotherapies, it remains to be determined how best to leverage the local immune landscape and/or optimally reprogram infiltrating cells towards pro-inflammatory phenotypes. In our recent publication, we explored magnifying the FR-B T cell + anti-PD-1 response through vaccine boosting, where improved tumor control was accompanied by increased accumulation of vaccine-boosted tumor-specific CD8+ T cells in the TME [4]. Alternatively, multiple groups have reported on ‘armoring’ approaches, wherein T cell therapies (eg. CAR T cells) are engineered to secrete proinflammatory cytokines to bolster the antitumor response [22–24].

## 4. Potential Opportunities to Exploit the Tumor Immune Landscape

There has been a concerted effort to better define predictive biomarkers of immunotherapy response, mechanistic drivers of immune escape, as well as strategies to rationally select combination immunotherapy approaches. Given inherent challenges related to intrinsic differences in the cellular composition of human and mouse TMEs [25] and (by extension) in effectively modeling clinical cancer treatments using preclinical models, analysis of serially collected patient samples at baseline and during immunotherapy treatment is likely to facilitate the development of improved ‘reverse translational’ models to effectively study clinical immunotherapy failure points using relevant lab-based assays. Importantly, such preclinical studies also provide an opportunity to carefully dissect and confirm the underlying mechanisms driving therapeutic response or resistance, strengthening the value of correlative observations linking unique tumor immune landscapes with response outcome, which should be interpreted with caution prior to definitive experimental evidence. Along with analysis of clinical samples using conventional assays, recent studies have demonstrated that incorporating advanced technologies to decipher spatial transcriptomic/proteomic signatures have the potential to uncover previously unappreciated immune subsets, novel immune cell contextures, as well as localized immune niches associated with productive antitumor responses [3,26,27]. Although the analysis of these large complex datasets can create fundamental challenges, multiple recent publications have successfully implemented the use of innovative AI and machine learning approaches to effectively integrate and analyze large single cell and spatial transcriptomic/proteomic datasets [28–32], with new analysis pipelines regularly emerging. While these new developments are likely to continue to provide novel insights related to the complex TME and inform previously unexplored therapeutic opportunities, such approaches require access to advanced technologies for generating the requisite input data, as well as the infrastructure and expertise needed to produce insightful conclusions. As such, these approaches may ultimately prove to be practical only in the setting of larger academically focused centers. Along with these technical limitations, patient welfare needs to be considered paramount, in addition to their willingness to consent to often invasive sample collection, particularly if done serially or if tumors are located in complex anatomical locations. This can in turn create additional challenges related to sampling bias, potential for heterogeneity in cellular composition or spatial organization within primary tumor lesions and when considering sampling from metastatic disease across anatomical locations, as well as variability in data generated across different platforms. Based on these current challenges, data collection and analysis pipelines may need to further mature before integration of larger ‘omics’ based data sets can be widely or practically applied to clinical cancer care, although current rates of innovation will undoubtedly speed up this process. However, it is important to also carefully consider contexts where adding further complexity to ‘hot versus cold’ classifiers would be practical or necessary, particularly in instances where clinically actionable decisions will not be impacted by access to additional data.

Collectively, integrating in-depth TME analyses that decipher key immunological features (or lack thereof) will permit tailored design of immunotherapies for cancer patients, with on treatment analysis of the TME supporting further refinement to enhance response durability (Figure 1D). As current and emerging immunotherapies continue to advance clinically, refined approaches of defining actionable immune compositions, states, or topographies that better predict outcomes, can be therapeutically leveraged, or which serve as early identifiers of responder/non-responder phenotypes will serve as an important complement to improving the lives of patients diagnosed with cancer.

## Figures and Tables

**Figure 1.**
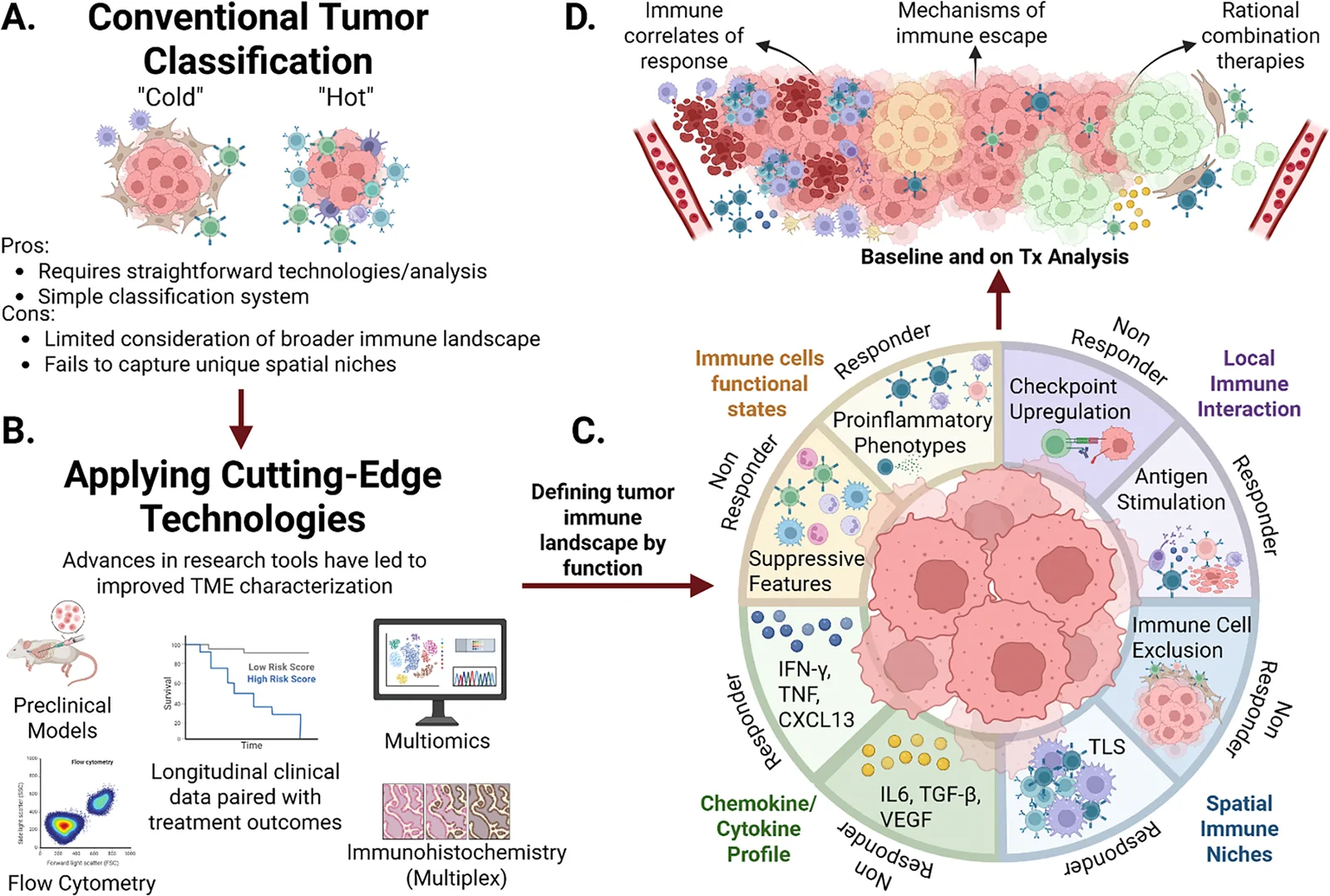
Reclassifying tumor immune landscapes through high resolution characterization. **(A-D)** Schematic overview of conventional and emerging approaches to improve the immune classification of the tumor microenvironment, as well as potential new knowledge that can be gained from defining immune heterogeneity with improved resolution. CXCL13: Chemokine (C-X-C Motif) Ligand 13; IFN-γ: Interferon Gamma; IL-6: Interleukin 6; TGF-β: Transforming Growth Factor Beta; TLS: Tertiary Lymphoid Structure; TME: Tumor Microenvironment; TNF: Tumor Necrosis Factor; Tx: Treatment; VEGF: Vascular Endothelial Growth Factor.

## Data Availability

Not applicable.

## Abbreviations

The following abbreviations are used in this manuscript:

αSMA: Alpha Smooth Muscle Actin
Arg-1: Arginase 1
CAFs: Cancer-Associated Fibroblasts
CAR T cell: Chimeric Antigen Receptor T cell
CCL19: Chemokine (C-C Motif) Ligand 19
CCL21: Chemokine (C-C Motif) Ligand 21
CLEC9A: C-Type Lectin Domain Family 9 Member A
CRC: Colorectal Cancer
CTLA-4: Cytotoxic T-Lymphocyte Associated Protein 4
CXCL10: Chemokine (C-X-C Motif) Ligand 10
CXCL13: Chemokine (C-X-C Motif) Ligand 13
DCB: Durable Clinical Benefit
DR: Durable Response
FAP: Fibroblast Activation Protein
FRα: Folate Receptor Alpha
FR-B T cells: FRα-Targeted Bispecific T cell Engager-Secreting T cells
HER2: Human Epidermal Growth Factor Receptor 2
ICI: Immune Checkpoint Inhibitor
IFN-γ: Interferon Gamma
IL-6: Interleukin 6
LCP: Local Cellular Programs
LTS: Long-Term Survivors
MMR: Mismatch Repair
mTLS: Mature Tertiary Lymphoid Structure
myCAFs: Myofibroblastic Cancer-Associated Fibroblasts
MYH11: Myosin Heavy Chain 11
NSCLC: Non-Small Cell Lung Cancer
OC: Ovarian Cancer
PD: Progressive Disease
PD-1: Programmed Cell Death Protein 1
PD-L1: Programmed Death Ligand 1
PARP: Poly(ADP-ribose) Polymerase
R: Responder
R+: Enhanced Responder
scRNA-seq: Single Cell RNA Sequencing
STS: Short-Term Survivors
STARLETS: Spatiotemporal Tumor Clone Evolution Tracking System
SLC2A1: Solute Carrier Family 2 Member 1
TAMs: Tumor Associated Macrophages
TCR: T Cell Receptor
TGF-β: Transforming Growth Factor Beta
TIL: Tumor Infiltrating Lymphocyte/T cell
TLS: Tertiary Lymphoid Structure
TMB: Tumor Mutational Burden
TME: Tumor Microenvironment
TNF: Tumor Necrosis Factor
TSME: Tumor Spatial Microenvironment
Tx: Treatment
VEGF: Vascular Endothelial Growth Factor

## References

[1] Goswami S, Pauken KE, Wang L, Sharma P. Next-generation combination approaches for immune checkpoint therapy. Nature Immunology. 2024;25(12):2186–2199. DOI 10.1038/s41590-024-02015-4 PMC13112085 39587347

[2] Borcoman E, Kanjanapan Y, Champiat S, Kato S, Servois V, Kurzrock R, Novel patterns of response under immunotherapy. Annals of Oncology. 2019;30(3):385–396. DOI 10.1093/annonc/mdz003 30657859

[3] Ma K, Wang L, Li W, Tang T, Ma B, Zhang L, Turning cold into hot: emerging strategies to fire up the tumor microenvironment. Trends in Cancer. 2025;11(2):117–134. DOI 10.1016/j.trecan.2024.11.011 39730243

[4] Chiello JL, Shaikh N, Jacobi J, Gaulin N, Santos G, Keck C, BiTE-secreting T cells rationally combine with PD-1 blockade and vaccine boosting to reshape antitumor immunity in ovarian cancer. Molecular Therapy. 2026;34(1):455–478. DOI 10.1016/j.ymthe.2025.09.047 PMC12766903 41029902

[5] Rosner S, Connor S, Sanber K, Zahurak M, Zhang T, Gurumurthy I, Divergent Clinical and Immunologic Outcomes Based on STK11 Co-mutation Status in Resectable KRAS-Mutant Lung Cancers Following Neoadjuvant Immune Checkpoint Blockade. Clinical Cancer Research. 2025;31(2):339–351. DOI 10.1158/1078-0432.CCR-24-2983 PMC11739779 39545922

[6] Shu DH, Ho WJ, Kagohara LT, Girgis A, Shin SM, Danilova L, Immunotherapy response induces divergent tertiary lymphoid structure morphologies in hepatocellular carcinoma. Nat Immunol. 2024;25(11):2110–2123. DOI 10.1038/s41590-024-01992-w PMC12042221 39455893

[7] Chen M, Ma P, Zhang Y, Wang D, Yu Z, Fu Y, Divergent tumor and immune cell reprogramming underlying immunotherapy response and immune-related adverse events in lung squamous cell carcinoma. J Immunother of Cancer. 2023;11(10):e007305. DOI 10.1136/jitc-2023-007305 PMC10603341 37857527

[8] Rosario SR, Long MD, Chilakapati S, Gomez EC, Battaglia S, Singh PK, Integrative multi-omics analysis uncovers tumor-immune-gut axis influencing immunotherapy outcomes in ovarian cancer. Nat Commun. 2024;15(1):10609. DOI 10.1038/s41467-024-54565-8 PMC11621351 39638782

[9] Cui X, Gu X, Li D, Wu P, Sun N, Zhang C, Tertiary lymphoid structures as a biomarker in immunotherapy and beyond: Advancing towards clinical application. Cancer Lett. 2025;613:217491. DOI 10.1016/j.canlet.2025.217491 39862919

[10] Peyraud F, Guegan J-P, Vanhersecke L, Brunet M, Teyssonneau D, Palmieri L-J, Tertiary lymphoid structures and cancer immunotherapy: From bench to bedside. Med. 2025;613:217491. DOI 10.1016/j.medj.2024.10.023 39798544

[11] MacFawn IP, Magnon G, Gorecki G, Kunning S, Rashid R, Kaiza ME, The activity of tertiary lymphoid structures in high grade serous ovarian cancer is governed by site, stroma, and cellular interactions. Cancer Cell. 2024;42(11):1864–1881.e1865. DOI 10.1016/j.ccell.2024.09.007 PMC12236807 39393357

[12] Varghese A, Hess SM, Chilakapati S, Conejo-Garcia JR, McGray AJR, Zsiros E. Tertiary lymphoid structures: exploring opportunities to improve immunotherapy in ovarian cancer. Front Immunol. 2025;16:1473969. DOI 10.3389/fimmu.2025.1473969 PMC12137288 40475770

[13] Hammerl D, Martens JWM, Timmermans M, Smid M, Trapman-Jansen AM, Foekens R, Spatial immunophenotypes predict response to anti-PD1 treatment and capture distinct paths of T cell evasion in triple negative breast cancer. Nat Commun. 2021;12(1):5668. DOI 10.1038/s41467-021-25962-0 PMC8476574 34580291

[14] Liao J-Y, Wang J, Li H, Liu Z, Tian Z, Lv X, Longitudinal tissue analysis reveals microenvironmental changes correlate with combined immunotherapy and targeted therapy response in metastatic breast cancer. J Immunother Cancer. 2025;13(10):e012629. DOI 10.1136/jitc-2025-012629 PMC12506462 41052881

[15] Li J, Lin P, Wang H, Tang Z, Yan X, Chen X, Pan-cancer analysis of spatial transcriptomics reveals heterogeneous tumor spatial microenvironment. Cell Rep Med. 2026;7(5):102751. DOI 10.1016/j.xcrm.2026.102751 PMC13198256 41999752

[16] Feng C, Yang Y, Li G, Fang Z, Yang B, Zhao F, Spatial tumor evolution panorama of ovarian cancer. Cell Rep Med. 2026;7(3):102666. DOI 10.1016/j.xcrm.2026.102666 PMC13006426 41850247

[17] Nelson BH, Hamilton P, Phung MT, Milne K, Harris B, Thornton S, Immunological and molecular features of the tumor microenvironment of long-term survivors of ovarian cancer. J Clin Invest. 2024;134(24). DOI 10.1172/JCI179501 PMC11645148 39470729

[18] Acha-Sagredo A, Andrei P, Clayton K, Taggart E, Antoniotti C, Woodman CA, A constitutive interferon-high immunophenotype defines response to immunotherapy in colorectal cancer. Cancer Cell. 2025;43(2):292–307.e297. DOI 10.1016/j.ccell.2024.12.008 39824178

[19] Wang L, Chu H, Chen D, Wei Y, Jia J, Li L, SLC2A1+ tumour-associated macrophages spatially control CD8+ T cell function and drive resistance to immunotherapy in non-small-cell lung cancer. Nat Cell Biol. 2026;28(2):338–348. DOI 10.1038/s41556-025-01840-5 PMC12904792 41501177

[20] Ma L, Xiong B, Liu M, Tan K. Cellular neighborhoods in cancer. Nat Cancer. 2026;7(1):16–28. DOI 10.1038/s43018-025-01107-w PMC13080512 41545713

[21] Peyraud F, Guégan J-P, Rey C, Lara O, Odin O, Del Castillo M, Spatially resolved transcriptomics reveal the determinants of primary resistance to immunotherapy in NSCLC with mature tertiary lymphoid structures. Cell Rep Med. 2025;6(2):101934. DOI 10.1016/j.xcrm.2025.101934 PMC11866545 39909044

[22] Castelli S, Wilson WV, Uslu U, Finck AV, Rommel PC, Assenmacher C-A, IL-9 signaling redirects CAR T cell fate toward CD8+ memory and CD4+ cycling states, enhancing antitumor efficacy. Immunity. 2026;59(1):195–212.e111. DOI 10.1016/j.immuni.2025.10.021 41274291

[23] Zuo Y, Vohwinkel DJ, Dong B, McDowell JR, Guzman BV, Manickavel Pandian TS, IL-36γ armored CAR T cells reprogram neutrophils to induce endogenous antitumor immunity. Cancer Cell. 2026;44(2):366–382.e310. DOI 10.1016/j.ccell.2025.11.007 PMC13007796 41386225

[24] Yagel G, Rimini D, von Locquenghien M, Avellino R, Barboy O, Chalan P, Tumor-antigen-independent targeting of solid tumors by armored macrophage-directed anti-TREM2 CAR T cells. Cancer Cell. 2026;44(3):519–533.e510. DOI 10.1016/j.ccell.2025.11.009 41576928

[25] Courau T, Jaszczak RG, Samad B, Flynn E, Chew NW, Reeder GC, Differential assembly of mouse and human tumor microenvironments. Nat Immunol;27(6):1282–1293. 2026. DOI 10.1038/s41590-026-02505-7 PMC13226090 42156893

[26] Aung TN, Monkman J, Warrell J, Vathiotis I, Bates KM, Gavrielatou N, Spatial signatures for predicting immunotherapy outcomes using multi-omics in non-small cell lung cancer. Nat Genet. 2025;57(10):2482–2493. DOI 10.1038/s41588-025-02351-7 PMC12513832 41073787

[27] Li X, Pan L, Li W, Liu B, Xiao C, Chew V, Deciphering immune predictors of immunotherapy response: A multiomics approach at the pan-cancer level. Cell Rep Med. 2025;6(4). DOI 10.1016/j.xcrm.2025.101992 PMC12047473 40054456

[28] Xing X, Cai L, Ouyang J, Wang F, Li Z, Liu M, Proteomics-driven noninvasive screening of circulating serum protein panels for the early diagnosis of hepatocellular carcinoma. Nature Commun. 2023;14(1):8392. DOI 10.1038/s41467-023-44255-2 PMC10728065 38110372

[29] Li Z, Li Y, Xiang J, Wang X, Yang S, Zhang X, AI-enabled virtual spatial proteomics from histopathology for interpretable biomarker discovery in lung cancer. Nature Med. 2026;32(1):231–244. DOI 10.1038/s41591-025-04060-4 PMC12823406 41491099

[30] Chen H, Deng C, Gao J, Wang J, Fu F, Wang Y, Integrative spatial analysis reveals tumor heterogeneity and immune colony niche related to clinical outcomes in small cell lung cancer. Cancer Cell. 2025;43(3):519–536.e5. DOI 10.1016/j.ccell.2025.01.012 39983726

[31] Aoki T, Duns G, Rai S, Jiang A, Lytle A, Yin Y, Multidimensional Characterization of Tumor–Immune Architecture Reveals Clinically Relevant Classic Hodgkin Lymphoma Subtypes. Cancer Discov. 2026;16(5):993–1013. DOI 10.1158/2159-8290.CD-25-0859 41615883

[32] Zhang W, Brown EL, Usmani A, Earland N, Kang M, Olelewe C, Non-invasive profiling of the tumour microenvironment with spatial ecotypes. Nature. 2026;654(8120):1076–1087. DOI 10.1038/s41586-026-10452-4 PMC13293879 42092150

